# Composite Nanofibers as Novel Sorbents for On-Line and Off-Line Solid-Phase Extraction in Chromatographic System: A Comparison for Detection of Free Biogenic Monoamines and Their Metabolites in Plasma

**DOI:** 10.3390/molecules27206971

**Published:** 2022-10-17

**Authors:** Liqin Chen, Yueling Bi, Tong Xu, Xiaohuan Li, Zhongze Fang

**Affiliations:** 1 Department of Toxicology and Sanitary Chemistry, School of Public Health, Tianjin Medical University, Tianjin 300070, China; 2 Tianjin Key Laboratory of Environment, Nutrition and Public Health, Tianjin 300070, China; 3 Department of Pharmacy, Tianjin Xiqing Hospital, Tianjin 300380, China

**Keywords:** on-line packed-fiber solid-phase extraction, nanofibers, HPLC, biogenic monoamines, plasma

## Abstract

Two different pretreatment approaches have been used for the enrichment and separation of biogenic monoamines and metabolites in plasma for high performance liquid chromatography (HPLC) determination. The first approach, based on on-line packed-fiber solid-phase extraction (PFSPE) coupled with HPLC, allows for the simultaneous detection of epinephrine (E), norepinephrine (NE), dopamine (DA), 3-methoxyl epinephrine (MN), norepinephrine (NMN), 3-methoxytyramine (3-MT), and 5-hydroxytryptamin (5-HT). Using this developed on-line PFSPE–HPLC method, the limit of detections (LODs) of the seven analytes ranged from 1 ng/mL (NMN and MN) to 2 ng/mL (NE, E, DA, 3-MT and 5-HT). The reportable ranges were 5–300 ng/mL for NE and DA, 5–100 ng/mL for E, and 5–200 ng/mL for NMN, MN, 3-MT and 5-HT. The off-line PFSPE–HPLC was employed in the second approach and could provide simultaneous detection of NE, E, DA, NMN, and MN. The linearity was verified in the range of 0.5–20 ng/mL (NE, E, and DA) and 20–250 ng/mL (NMN and MN). The LODs of the five analytes ranged from 0.2 ng/mL (NE, E, and DA) to 5 ng/mL (NMN and MN). This study verified the possibility of using nanofibers as an adsorbent in an on-line PFSPE–HPLC system for the determination of biogenic monoamines and their metabolites in human plasma. Compared with the off-line PFSPE approach, the on-line PFSPE method deserves attention mainly due to its greener character, derived from the automation of the process and high-throughput with less operators’ handling.

## 1. Introduction

Sample preparation is an important step towards isolating the desired compounds from complex matrices and eventually influences the reliability and accuracy of the whole analysis. After years of development of sample pretreatment techniques, there are many different pretreatment methods, such as liquid–liquid microextraction (LLME), dispersive liquid–liquid microextraction (DLLME), solid-phase extraction (SPE) and solid-phase microextraction (SPME) [1,2,3,4,5]. Among them, DLLME and SPME have been identified as green sample pretreatment techniques and have greatly improved sample pretreatment quality and efficiency. However, the use of organic solvents, high costs, short life usability, and long extraction times are the main disadvantages of these techniques. Therefore, automation, miniaturization, environmental protection, and high efficiency are always the future development trends of sample pretreatment techniques [6]. New and better sample pretreatment methods still need to be developed.

Packed-fiber solid-phase extraction (PFSPE) is an efficient and simple pretreatment method, developed in 2007 [7]. The technique has long been processed off-line, which requires manual pushing of the sample to be processed through the nanofiber extraction column. Finally, the adsorbed targets were eluted with a small amount of solvent (about 100 μL), which could be directly injected into the instrument for analysis without nitrogen blowing and concentration. This off-line PFSPE processing mode is simple and easy to operate and has been applied in many studies [8,9,10,11,12], showing the great potential of nanofibers as novel adsorbents in the field of sample pretreatment. With the rapid development of this technique, increasing laboratories have begun to acknowledge this novel sample pretreatment method and use it in a series of related analytical tests [13,14,15]. However, the additional requirements for laboratory operators of off-line operation hinder the further promotion and application of this technique.

The PFSPE technique has been available for on-line processing since 2018 [16]. Czech scholars used the on-line coupling of nanofibrous extraction with column-switching to detect bisphenol A in environmental water samples, which verified the possibility of using nanofiber as an on-line SPE material. Since then, the on-line PFSPE technique has begun to undergo rapid development, and various nanofibers, detection matrices, and target compounds have appeared in the field of on-line PFSPE research [17,18,19,20,21,22]. In our previous work, a method of on-line PFSPE using polycrown ether composite nanofibers was also developed to determine the number of catecholamines (CAs) in urine sample [23]. An on-line PFSPE column packed with 10 mg nanofibers can be reused over 100 times, showing the excellent stability and repeatability of the adsorbents. Compared with the off-line operation, using 2–3 mg nanofibers at a time, the service efficiency of the on-line PFSPE column is improved 100 times, and material resources are greatly saved.

The purpose of this study is to expand the number of previous research analytes, including CAs’ metabolites and 5-hydroxytryptamin (5-HT), in the research scope, compare the advantages and disadvantages of the on-line PFSPE and off-line PFSPE methods, and accumulate more research experience for the further development of the PFSPE technique. Biogenic monoamines include CAs and indole substances (5-HT is an indole representative substance). CAs are typical representatives of a class of highly polar compounds, which mainly include epinephrine (E)), norepinephrine (NE), and dopamine (DA). Their 3-methoxyl metabolites, including 3-methoxyl epinephrine (MN), norepinephrine (NMN), and 3-methoxytyramine (3-MT), have also been commonly used indicators in recent studies [24,25,26]. CAs and 5-HT, which play an important role in the physiological function of human body and are closely related to human health, are important neurotransmitters in the central system.

Biogenic monoamines have good natural fluorescence. On the basis of previous experiments, this experiment also uses the polycrown ether nanofibers to achieve the enrichment and separation of biogenic monoamines and metabolites in plasma samples by optimizing the on-line PFSPE procedure. Consequently, a method for the detection and analysis of biogenic monoamines and metabolites in plasma samples by on-line PFSPE HPLC system coupling with fluorescence detector was established. Moreover, through the comparison of the two pretreatment methods, on-line PFSPE and off-line PFSPE, this study further highlights the possibility and necessity of developing the on-line PFSPE methods.

## 2. Results and Discussion

### 2.1. Optimization of the On-Line PFSPE Procedure

The tested parameters of the on-line PFSPE extraction were the composition and the flow rate of the separation mobile phase and the carrier mobile phase and the duration of the extraction step.

#### 2.1.1. Mobile Phase Gradient Elution Procedure

**Figure 1:**
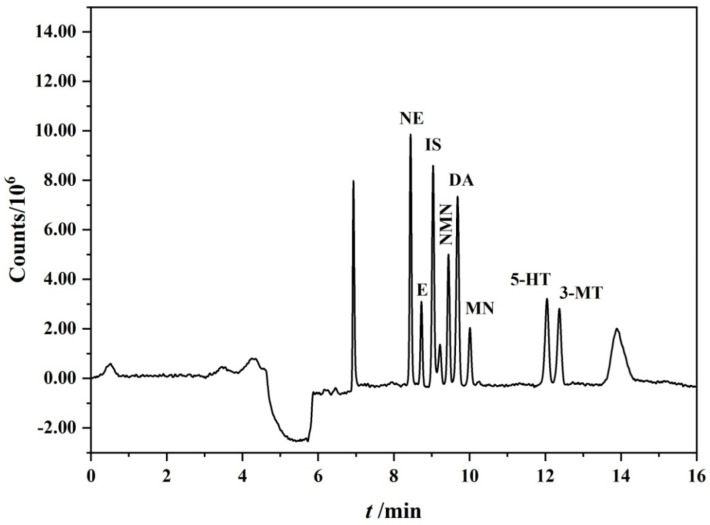
The chromatogram of on-line enrichment and separation of the biogenic monoamines and metabolites.

The composition of the separation mobile phase was the first tested parameter. Isometric elution can separate CAs well, but when the metabolites of CAs and 5-HT were added, it was found that 3-MT and 5-HT were difficult to separate, and their retention times were almost the same. Therefore, gradient elution was tried for separation. After several attempts, the two substances could be separated by a gradient elution design between the mobile phases A and B. Consequently, Table 1 shows the best mobile phase condition for the separation of the biogenic monoamines and metabolites. The chromatogram of the on-line enrichment and separation of these eight substances under the optimal condition is shown in Figure 1.

#### 2.1.2. Optimization of the Duration of the On-Line Extraction Step

**Figure 2:**
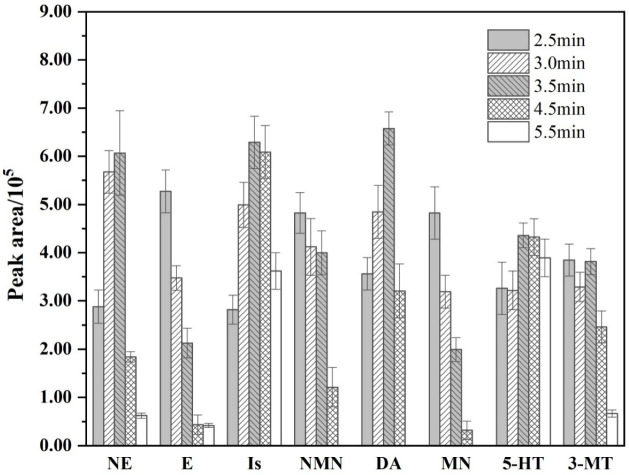
Comparison of peak area of the biogenic monoamines at different durations.

The duration is a very important factor in the on-line procedure, so the effect of duration on the targets was also studied in this experiment. Five kinds of duration were tested from 2.5 min to 5.5 min. As shown in Figure 2, with the increase in duration, the peak area of most target substances showed a trend of first increasing and then decreasing. Moreover, some target substances fused with impurity peaks after a duration over 4.5 min, and the resolution was not good (shown in Figure S1). Meanwhile, the peak area of DPBA decreases with the increase in duration, and the separation from adjacent target substances becomes better, which can avoid the interference of adjacent target substances. Therefore, considering the separation and response values of biogenic monoamines and impurity peaks, the duration at 3.5 min was chosen as the best duration of the on-line extraction step.

#### 2.1.3. Optimization of Carrier Mobile Phase for Left Pump

**Figure 3:**
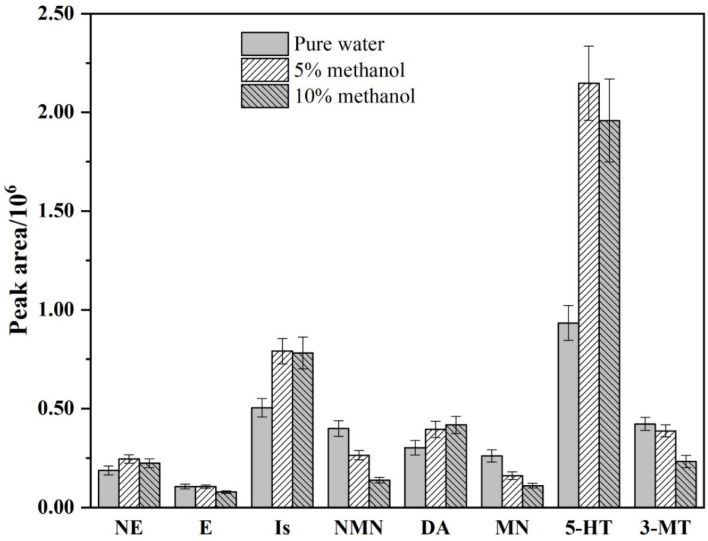
Comparison of peak area of the biogenic monoamines at different carrier mobile phase.

The biogenic monoamines are a kind of highly polar compound. The polarity of the carrier mobile phase for the left pump will affect the enrichment effect of the biogenic monoamines on the PFSPE column and the separation of the interfering impurities. Therefore, the composition of the carrier mobile phase was the third tested parameter. The mixtures of the methanol and water were tested at concentrations ranging from 0% to 20%. Figure S2 shows that with the change of the carrier mobile phase composition, the separation and response of the target substance both have some changes. Especially when the proportion of methanol reached 20%, the fusion between NMN and the previous impurity peak had occurred, and the signal exceeded the standard during the peak period of the solvent peak. Hence, only the response values of the biogenic monoamines and metabolites under the three methanol water proportions are counted in Figure 3. According to the statistical results, the response values of CAs and 5-HT increased with the increase in the proportion of methanol in the carrier solution, while the trend of the metabolites was the opposite. Adding a certain proportion of methanol solution in the carrier mobile phase is conducive to the removal of impurities in the real biological sample. As a result, the carrier mobile phase containing 5% methanol in water is selected as the best carrier mobile phase, after comprehensive consideration.

### 2.2. Influence of Sample Enrichment Volume of the On-Line PFSPE Procedure

**Figure 4:**
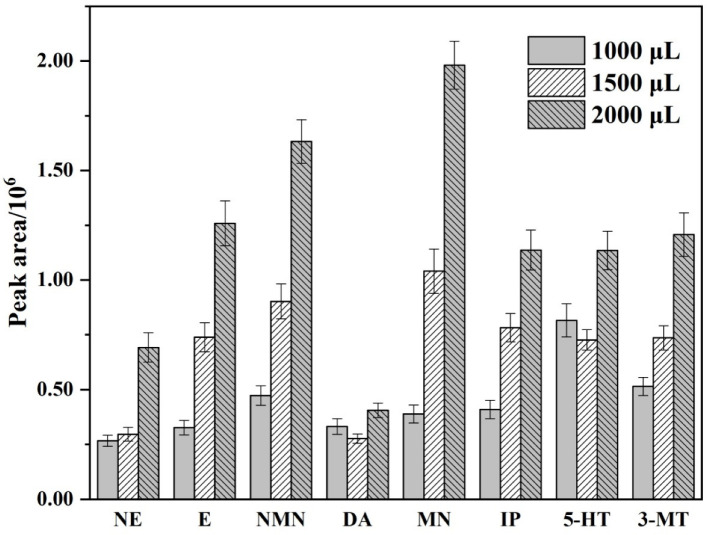
Comparison of peak area of the biogenic monoamines at different injection volume.

This experiment was equipped with an autosampler with a maximum injection volume of 2500 μL, and the effect of the sample volume was mainly investigated in the large volume range of 1000 to 2500 μL (Figure S3 shows the chromatogram of different sample enrichment volumes). As Figure 4 shows, the extraction efficiency increased up to 2000 μL, and it started to exceed the signal range when reaching 2500 μL. Moreover, an interesting phenomenon was found: the signal response of E, NMN, and MN would change dramatically when the injection volume increased to 2000 μL. Their signal values increased 3.9-fold, 3.5-fold, and 5.1-fold, respectively, compared with 1000 μL of injection volume. Therefore, according to the experimental phenomenon, increasing the injection volume to about 2000 μL should contribute to the enrichment efficiency of these target substances. Consequently, an injection volume of 2000 µL was chosen as the optimal possible volume.

### 2.3. Establishment of An On-Line PFSPE–HPLC Method for Detecting Plasma Samples

After protein precipitation and pH neutralization, the volume of a 500 μL plasma sample is approximately 800 μL, and up to 600 μL can be injected (the sampler cannot completely aspirate the sample). It was found that the response values of the target substances were very low, especially those of NMN, MN, 5-HT, and 3-MT, which were almost undetected by injecting 600 μL of 100 ng/mL spiked plasma after two-step treatment. In order to improve the detection limit of this method, the approaches of diluting the treated plasma samples with water and increasing the injection volume were both tried. The results showed that the response values of each target substance increased significantly, especially for the four undetected substances mentioned above, which could also be at the same level as the CAs’ values. However, with the injection volume increased, the impurity peak next to the original IS also exceeded the signal range, resulting in the position of NE and E just on the peak slope platform, and the signal of the original IS was obscured. Then, in this case, the internal standard can only be replaced, and another internal standard IP was adopted. The peak position of IP was between the target substances, just to avoid the interference of the impurity peak. Therefore, the internal standard adopted was replaced by IP under the on-line PFSPE–HPLC method.

**Figure 5:**
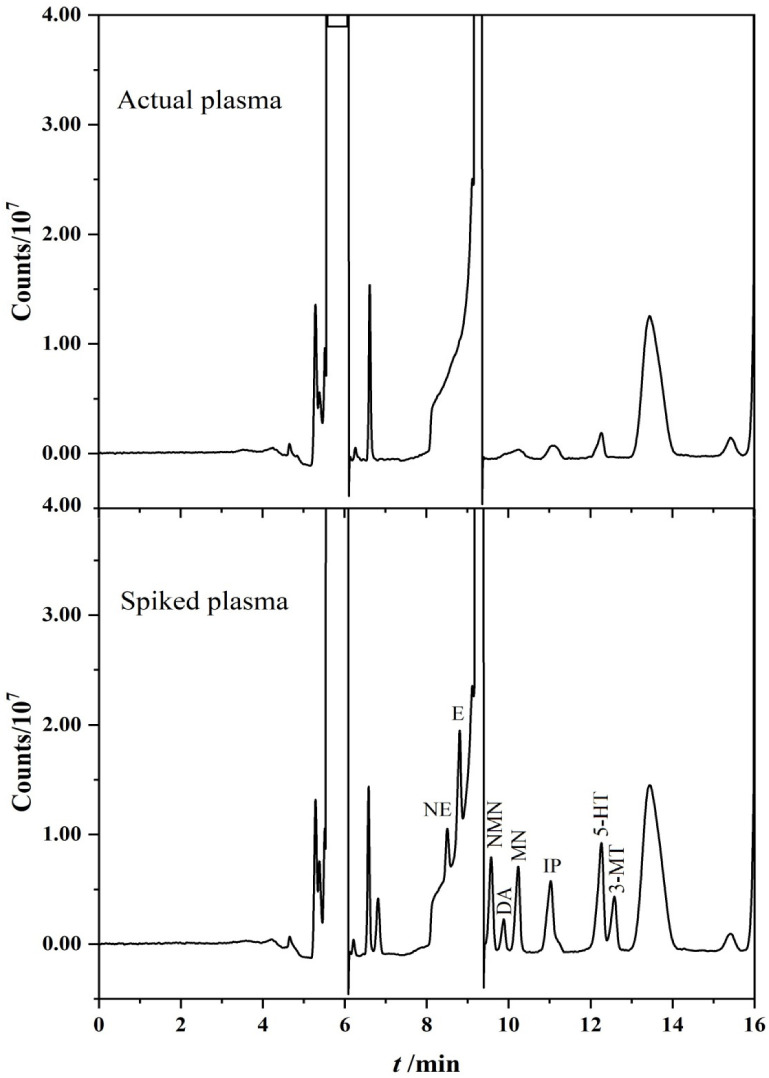
Chromatograms of spiked plasma (100 ng/mL) and actual plasma using on-line PFSPE–HPLC method.

The volume of water added to the treated plasma sample was determined to be 900 μL, which ensured an injection volume of 1500 μL. It can be seen from the discussion under 3.2 that the injection volume of 2000 μL is better than 1500 μL, but the influence of the impurity peak should be taken into account in the real sample detection. Depending on the experimental situation, when the injection amount is greater than 1500 μL, the response value of the target substances does increase, but the peak broadening of the impurity peak is more serious, which will cover the signal of NMN peak at the same time. Therefore, in order to give consideration to the detection of more target substances, it is appropriate to choose the injection volume of 1500 μL, and the volume of water added is determined as 900 μL. Figure 5 shows the chromatograms of actual plasma and spiked plasma using on-line PFSPE–HPLC method.

### 2.4. Linearity and Recovery of the On-Line PFSPE–HPLC Method

The method linearity of the on-line PFSPE–HPLC was tested by spiked plasma in the range of 5–300 ng/mL using seven calibration points. After treatment, 900 μL of water was added to the spiked plasma and mixed, and 1500 μL was injected into the HPLC system for further on-line pretreatment and analysis. The calibration curves were constructed by plotting the peak area ratio of the targets-to-IP against the concentrations of the targets. The linearity of NE and DA was ideal within the range of 5–300 ng/mL, while E is at a higher peak position, so the upper linear limit is affected and becomes linear in the range of 5–100 ng/mL. The linearity of NMN, MN, 3-MT, and 5-HT was ideal within the range of 5–200 ng/mL. The limit of detection (LOD) for NE, E, DA, 3-MT, and 5-HT was 2 ng/mL (S/N = 3), and the limit of detection (LOD) for NMN and MN was 1 ng/mL (S/N = 3). The parameters of the calibration curves, including the slope, intercept, and regression coefficient, are presented in Table 2.

Method accuracy was evaluated by comparing the percentage of recovery to the standard addition, using 100 ng/mL standards in real plasma samples. As a completely clean plasma matrix was impossible to obtain, recovery was calculated by subtracting the response from the blank matrix after correction with IP. Recovery was found to be 93.4 ± 3.3, 86.1 ± 1.5, 119.4 ± 2.4, 90.8 ± 5.4, 98.4 ± 3.4, 109.8 ± 5.7, and 106.3 ± 5.5 for NE, E, DA, NMN, MN, 3-MT, and 5-HT, respectively. The intraday precision of the method was tested using a spiked plasma sample at a concentration of 100 ng/mL. It was injected six times. The values of the intraday precision were below 8.0%.

### 2.5. Establishment of an Off-Line PFSPE–HPLC Method for Detecting Plasma Samples

**Figure 6:**
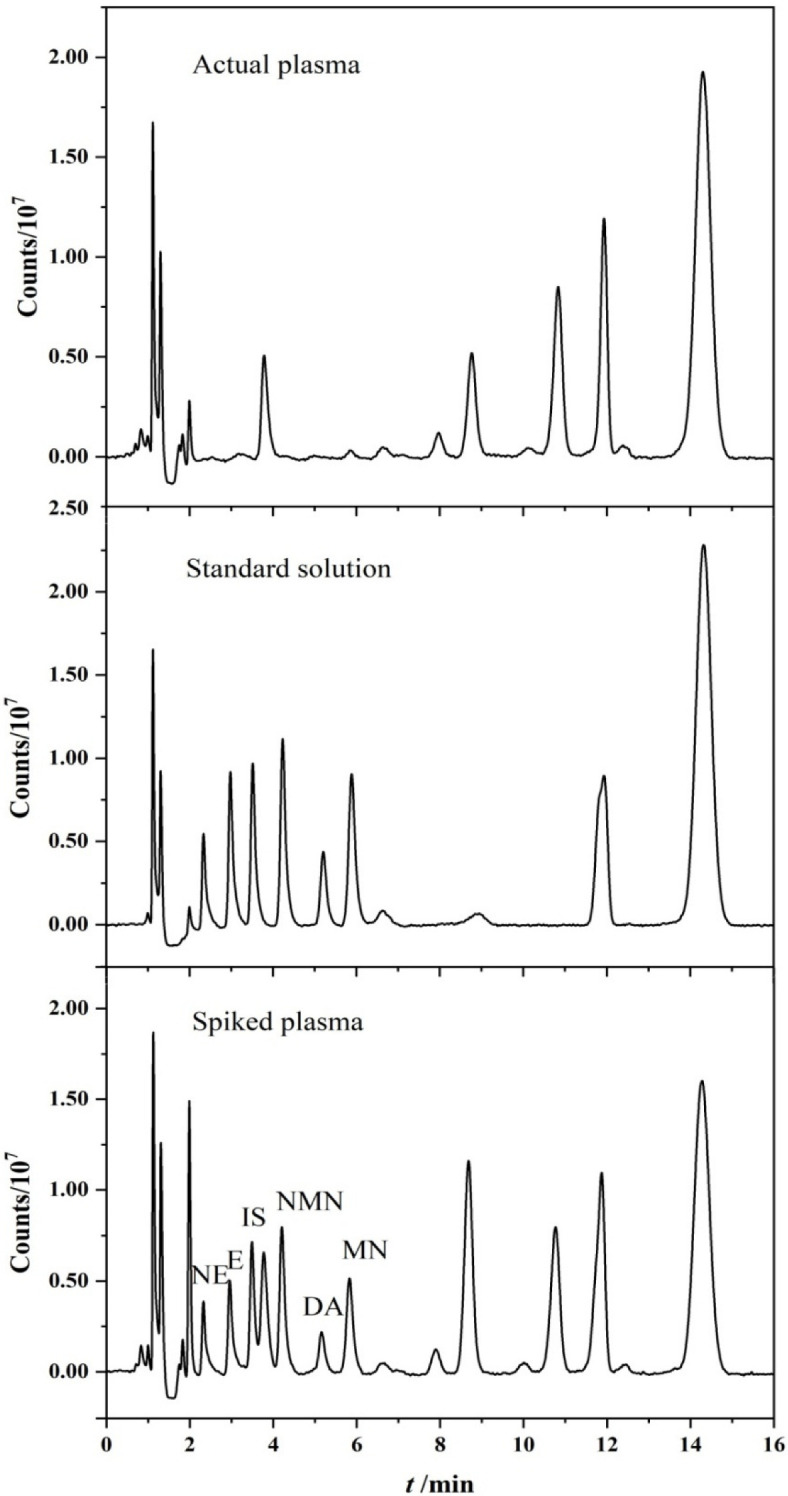
Chromatograms of spiked plasma (10 ng/mL of CAs and 200 ng/mL of NMN and MN), standard solution (10 ng/mL) and actual plasma using off-line PFSPE–HPLC method.

In the off-line working mode, the gradient elution method was used to find that the peaks of the target substances were extremely unstable. In order to avoid the influence of peak fluctuation on the accuracy of the method, the isocratic elution method was finally used to separate the target substances. However, 3-MT and 5-HT could not be separated under this condition, so only five target substances were analyzed in the off-line method. In blank plasma, an impurity peak appeared next to IS. However, this impurity peak could be separated from IS in the chromatogram of the spiked plasma without affecting the analytical results. Figure 6 shows the chromatograms of actual plasma, standard solution and spiked plasma using off-line PFSPE–HPLC method.

### 2.6. Linearity and Recovery of the Off-Line PFSPE–HPLC Method

The linearity of the off-line PFSPE–HPLC method was tested by spiked plasma in the range of 0.5–20 ng/mL (NE, E, and DA) and 20–250 ng/mL (NMN and MN), using seven calibration points. The calibration curves were constructed by plotting the peak area ratio of the targets-to-IS against the concentrations of the targets. The linearity of NE, E, and DA was ideal within the range of 0.5–20 ng/mL, while the linearity of NMN and MN was ideal within the range of 20–250 ng/mL. The limit of detection (LOD) for NE, E, and DA was 0.2 ng/mL (S/N = 3), and the limit of detection (LOD) for NMN and MN was 5 ng/mL (S/N = 3). The parameters of the calibration curves, including the slope, intercept, and regression coefficient, are presented in Table 3.

The accuracy parameter was evaluated by the determination of the recovery using a standard addition procedure, with the plasma samples spiked at the concentration level (10 ng/mL for NE, E, and DA and 100 ng/mL for NMN and MN), each in triplicate. The obtained recovery values are also shown in Table 3.

### 2.7. Comparison of the Off-Line and On-Line PFSPE–HPLC Methods

The main aim of the presented work was to demonstrate the possibility to use nanofibers as an adsorbent in an on-line PFSPE–HPLC system, for the determination of the biogenic monoamines and their metabolites in plasma. The developed method is a fully automated procedure using PDB18C6/PS composite nanofibers for the extraction of biogenic monoamines and their metabolites in plasma. The previous work, utilizing the PDB18C6/PS composite nanofibers for the off-line PFSPE followed by HPLC with ECD detection for the determination of CAs in plasma, was published in 2016 [27]. In this study, an off-line PFSPE–HPLC–FLD method was used to extend the analysis of the two metabolites, with a limit of detection of 5 ng/mL. Both off-line PFSPE methods have a 0.2 ng/mL limit of detection for CAs. In contrast, the detection limit of CAs for the on-line PFSPE–HPLC method is not as good as that for the off-line PFSPE–HPLC method, with a difference of 10 times. However, the detection limit of metabolites by the on-line PFSPE–HPLC method is superior to that by the off-line PFSPE–HPLC method. Moreover, the on-line PFSPE–HPLC method provides simultaneous enrichment detection of more target substances. Table 4 lists the working patterns of the two approaches and their respective advantages and disadvantages.

The developed on-line PFSPE–HPLC method is highly automated, and the high-pressure fluid can easily rebalance the nanofibers for multiple uses. From our data, it is known that the number of reuses can be as high as 200 or more, and even up to 100 or more times for biological samples. However, the PFSPE column used in the off-line method can only be used once due to the high loading pressure, which makes it difficult to be reused, not to mention the high requirements of the off-line PFSPE–HPLC method for the laboratory operators and the heavy labor that is involved. However, the on-line PFSPE–HPLC method has no such difficulties and can be said to reduce the labor of the experimental operators to a large extent. Therefore, although there are still some gaps in the detection limit of CAs, the on-line PFSPE–HPLC method has more advantages in the detection limit of metabolites and determination of more analytes, and can be reused hundreds of times with high throughput and less operators’ handling. These advantages will eventually show the great potential of the on-line PFSPE–HPLC method in the field of sample pretreatment in the future.

## 3. Experimental

### 3.1. Materials

Epinephrine hydrochloride (E), norepinephrine bitartrate (NE), dopamine hydrochloride (DA), 3,4-dihydroxybenzylamine hydrobromide (DHBA), metanephrine (MN), normetanephrine (NMN), and 3-methoxytyramine (3-MT), serotonin (5-HT), isoprenaline (IP), and diphenylborinic acid 2-aminoethyl ester (DPBA) were purchased from Sigma-Aldrich (St. Louis, MO, USA). HPLC–grade methanol and acetonitrile were obtained from Tianjin Chemical Reagent Company (Tianjin, China). All other reagents were of analytical grade, unless otherwise indicated. Polystyrene (PS) (Mw = 185,000) was provided by Shanghai Chemical Agents Institute (Shanghai, China). Poly(dibenzo-18-crown-6) (PDB18C6) was synthesized in the laboratory of Tianjin Medical University [28]. An empty 10 × 2.1 mm column cartridge kit was purchased from Dalian Replete technology instrument Co., Ltd. (Dalian, China).

### 3.2. Instrumentation

HPLC with a fluorescence detector was carried out on an UltiMate3000 HPLC connected to an FLD–3100 fluorescence detector (Thermo Scientific, Waltham, MA, USA). The wavelengths of excitation and emission were 286 and 318 nm, respectively. Samples were injected into a YMC–Pack ODS–A column (100 × 4.0 mm, 3 μm particle size) via a WPS-3000 SL autosampler (Maximum quantitative ring volume 2500 μL). An HPLC software package (Chromeleon 7.2 SR5) was used for the data analysis. The mobile phase for left pump was 5% methanol. The mobile phase of A for right pump was 1 mol/L acetic acid (HOAc). The mobile phase of B for right pump consisted of 5.5% acetonitrile, 45 mM sodium dihydrogen phosphate, 35 mM citric acid, 2 mM sodium heptanesulfonate, and 0.25 mM EDTA. The pH was adjusted to 4.2 by 2 M NaOH, and the flow rate was set at 0.5 mL/min. The temperature of the column oven was set to 35 °C.

### 3.3. Preparation of Standard Solutions and Samples

Solutions (1 mg/mL) of CAs, NMN, MN, 3-MT, 5-HT, IP, and DHBA were prepared by dissolving appropriate amounts of respective chemicals in water and storing them in the dark at −20 °C. DPBA solution (2.0 mg/mL) was prepared, as mentioned above, and used as a complexing reagent.

### 3.4. Preparation of Electrospun Nanofibers

The PDB18C6/PS composite nanofibers were fabricated by electrospinning, as described elsewhere [28]. Then, 15% PS and 5% PDB18C6 were mixed and electrospun to prepare a composite nanofiber for adsorbent in this study. Related material characterization can be found in the literature [28].

### 3.5. Preparation of Column for On-Line PFSPE and Off-Line PFSPE

The extraction columns for on-line PFSPE were prepared manually by packing the PDB18C6/PS composite nanofibers (about 10 mg) into an empty column cartridge (10 × 2.1 mm) with two removable sieve plates. The cartridge was placed in a guard column holder and connected to the system using HPLC fittings. Figure S4 in the Supplementary Materials shows the preparation of the on-line PFSPE column and connection to the HPLC system. Figure S5 shows the schematic diagram of on-line sample pretreatment. Figure S6 shows the schematic diagram of on-line transfer of the target compounds. The on-line PFSPE column was washed with 100% methanol for 4 min at a flow rate of 0.5 mL/min. Then, it was washed with ultrapure water for 5 min before use. 

A 1-mL microcolumn was used as off-line PFSPE column. The PFSPE columns were prepared as described in a previous paper [29]. Filter support was not necessary for packing fiber (3 mg) into a 1-mL microcolumn. The off-line PFSPE column was firstly preconditioned by 100 μL of methanol and 100 μL of water before use. 

### 3.6. Sample Preparation

Human plasma samples were obtained from healthy volunteers. The study protocol was approved by the Ethics Committee of Tianjin Xiqing Hospital, and all experimental procedures were conducted following the Declaration of Helsinki. All plasma samples were kept at −80 °C until analysis. Then, 5 μL of DHBA (internal standard IS for off-line procedure, 1 μg/mL; IP for on-line procedure, 10 μg/mL) were added to 500 μL of plasma sample, and the plasma mixture was vortex-mixed and 100 μL trichloroacetic acid was added for protein deposition. After vortex-mixing and centrifuging at 10,000 rpm for 10 min, the supernatant (about 400 μL) was transferred to another tube. Next, 20 μL of 2.0 mg/mL DPBA, used as a complexing reagent, was added to the supernatant. The mixture was vortex-mixed and adjusted to pH 7.0 with phosphate buffer (pH 8) containing 2 M sodium hydroxide (80:5, *v*/*v*; approximately 400 μL), for further extraction.

### 3.7. Extraction Experiment of Off-Line PFSPE

Off-line PFSPE working conditions refer to the previous article [27]. The sample (the spiked water sample or the supernatant of spiked plasma sample after pH adjustment) was loaded onto the conditioned off-line PFSPE column and pushed through the sorbent by the pressure of air forced by a gastight plastic syringe (5 mL). The flow was carefully controlled in a slow dropwise manner. Then, the adsorbed targets were washed with 100 μL of water and eluted with 50 μL of 6 mol/L HOAc solution. Water (50 μL) was added into the eluent and vortex-mixed. Finally, 50 μL of the eluate mixture was injected into the HPLC column via an autosampler. 

### 3.8. HPLC Analysis and On-Line Column Switch Analysis

HPLC analysis for off-line PFSPE procedure used isocratic elution with the mobile phase B for right pump. The injection volume was 50 μL. The whole run time was 16 min.

The on-line PFSPE procedure was implemented by employing an on-line PFSPE column on the 10-port valve connected to an HPLC sampling loop located on a 6-port valve. The 10-port valve was used to design a procedure for on-line pretreatment and analysis. The first step was on-line sample pretreatment; in this step, the sample was loaded by an autosampler into the on-line PFSPE column for extraction and purification. Secondly, elution and transfer of the target compounds to the analytical column were carried out by switching the 10-port valve (straight-flushing). The third step involved separation and determination of the biogenic monoamines and metabolites, and the 10-port valve was switched back at this time to equilibrate the PFSPE column for the next run. The total running time was 16 min. Figures S5 and S6 show the schematic diagram of on-line sample pretreatment and transfer of the target compounds. 

A 1500 μL aliquot of sample was injected into the on-line PFSPE column. When the biogenic monoamines and metabolites were preconcentrated on the PFSPE column, the analytical column was simultaneously equilibrated with the mobile phase. The valve switch time was set to be the same as the extraction time at 3.5 min. Isocratic elution with the 1 mol/L HOAc lasted for 1 min to transfer the adsorbed targets to the analytical column. After that, the valve was switched back to balance the PFSPE column again, and a gradient elution program for right pump was used for separation and determination of the biogenic monoamines and metabolites at the same time. 

## 4. Conclusions

This work describes the first application of two pretreatment techniques, namely on-line PFSPE and off-line PFSPE, for the HPLC trace determination of biogenic monoamines and their metabolites in plasma. We have proven that the PDB18C6/PS composite nanofibers prepared by an electrospinning procedure are suitable for a high-pressure on-line PFSPE–HPLC system and add several benefits over the off-line PFSPE procedure for the determination of biogenic monoamines and their metabolites in plasma. One of the advantages of the on-line PFSPE–HPLC method is the ability to detect more monoamines simultaneously, especially in terms of the detection limit of the metabolites. Another important advantage of the use of the on-line PFSPE–HPLC is the prospect of repeated use of the extraction-column-packed nanofibers and the simplicity of the extraction-column-packing procedure. It can be said that the on-line PFSPE–HPLC method not only liberates the hands of the operator but also reduces the strict requirements for the operator during the packing and passing through of the column. Here, we demonstrated that nanofibers show a great potential in future use as new and modern extraction sorbents in the on-line PFSPE–HPLC system. Therefore, we believe that future research will emerge with more on-line PFSPE–HPLC methods to replace off-line PFSPE–HPLC methods.

## Figures and Tables

**Table 1:** The optimization procedures for on-line separation and enrichment of the biogenic monoamines and metabolites.

Time	Flow	Left Pump	Right Pump		Valve Position
(min)	(mL/min)	A (%)	A (%)	B (%)	
0	0.5	100	0	100	10_1
1	0.5	100	0	100	10_1
1.5	0.5	100	100	0	10_1
3	0.5	100	100	0	10_1
3.5	0.5	100	100	0	1_2
4.5	0.5	100	100	0	10_1
5	0.5	100	85	15	10_1
15	0.5	100	40	60	10_1
16	0.5	100	0	100	10_1

**Table 2:** Analytical performance of the developed on-line PFSPE–HPLC method for detection of biogenic monoamines and their metabolites in plasma.

Analyte	t_R_ (min)	Linear Range (ng/mL)	Regression Equation	r	LOD (ng/mL)	Amount Added (ng/mL)	Accuracy (%)	RSD (%)
NE	8.44	5–300	y = 0.0032x + 0.001	0.999	2	100	93.4	3.3
E	8.74	5–100	y = 0.0069x − 0.0114	0.998	2	100	86.1	1.5
DA	9.65	5–300	y = 0.0031x − 0.0027	0.994	2	100	119.4	2.4
NMN	9.42	5–200	y = 0.0079x − 0.017	0.999	1	100	90.8	5.4
MN	9.97	5–200	y = 0.0079x + 0.0066	0.999	1	100	98.5	3.4
3-MT	12.22	5–200	y = 0.0061x + 0.0021	0.999	2	100	109.8	5.7
5-HT	11.89	5–200	y = 0.011x − 0.0733	0.997	2	100	106.3	5.5

**Table 3:** Analytical performance of the developed off-line PFSPE–HPLC method for detection of biogenic monoamines and their metabolites in plasma.

Analyte	t_R_ (min)	Linear Range (ng/mL)	Regression Equation	r	LOD (ng/mL)	Amount Added (ng/mL)	Accuracy (%)	RSD (%)
NE	2.31	0.5–20	y = 0.0361x + 0.0442	0.998	0.2	10	107.9	1.2
E	2.94	0.5–20	y = 0.0508x + 0.0188	0.997	0.2	10	104.2	4.4
DA	5.11	0.5–20	y = 0.0387x − 0.0044	0.999	0.2	10	105.8	2.5
NMN	4.77	20–250	y = 0.0046x − 0.0015	0.997	5	100	108.3	5.8
MN	5.76	20–250	y = 0.0025x − 0.0164	0.995	5	100	107.6	8.9

**Table 4:** Comparison of the off-line and on-line PFSPE–HPLC methods.

Methods	Off-Line PFSPE	On-Line PFSPE
Schematic diagram	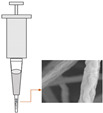	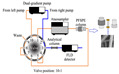
Process mode	Manual handling	Automation
Experience of operator	Takes effort to pass through the column	Without operation
Service efficiency	Single use	100 or more times
Amount of adsorbent	3 mg	10 mg
Analytes in this study	5 (NE, E, DA, NMN, and MN)	7 (NE, E, DA, NMN, MN, 5-HT, and 3-MT)
Limit of detection	CAs/0.2 ng/mL NMN and MN/5 ng/mL	CAs, 5-HT, and 3-MT/2 ng/mL NMN and MN/1 ng/mL
High throughput	Difficult	Easy
Enrichment factor ^a^	NE 5.7, E 5.1, NMN 4.6, DA 5.1, and MN 4.2	NE 5.4, E 5.1, NMN 4.3, DA 2.6, and MN 4.8
Requirements for operator	Higher requirements, especially for packing and passing through the column; otherwise, easy to affect the precision and accuracy	Packing the column is easy, only required for preparing samples
Establishment time	2007 [7]	2018 [16]
Application	Wide range of applications	Just started, so not many applications

^a^ Enrichment factor is defined as the ratio of peak area obtained by the PFSPE mode to that of the direct injection.

## Data Availability

The data presented in this study is available in Supplementary Material.

## Supplementary Materials

The following supporting information can be downloaded at: https://www.mdpi.com/article/10.3390/molecules27206971/s1. Figure S1: The chromatogram of different duration of the on-line extraction step; Figure S2: The chromatogram of different carrier mobile phase of the on-line extraction step; Figure S3: The chromatogram of different sample enrichment volume of the on-line extraction step; Figure S4: The preparation of the on-line PFSPE column and connection to the HPLC system; Figure S5: The schematic diagram of on-line sample pretreatment; Figure S6: The schematic diagram of on-line transfer of the target compounds.
