# Supplementary material for: Composite Nanofibers as Novel Sorbents for On-Line and Off-Line Solid-Phase Extraction in Chromatographic System: A Comparison for Detection of Free Biogenic Monoamines and Their Metabolites in Plasma
Source: Molecules. 2022 Oct 17;27(20):6971. doi: 10.3390/molecules27206971 (PMC9611131; doi:10.3390/molecules27206971)
Supplement: Supplementary file 1 [file molecules-27-06971-s001.zip › molecules-1961205-supplementary.pdf]

Composite nanofibers as novel sorbents for on-line and off-line solid phase extraction in chromatographic system: A comparison for detection of free biogenic monoamines and their metabolites in plasma

Liqin Chen [1,2,*,†], Yueling Bi [3,†], Tong Xu [1], Xiaohuan Li [1] and Zhongze Fang [1,2,*]

***** Correspondence: chenliqin@tmu.edu.cn (L.C.); fangzhongze@tmu.edu.cn (Z.F.)

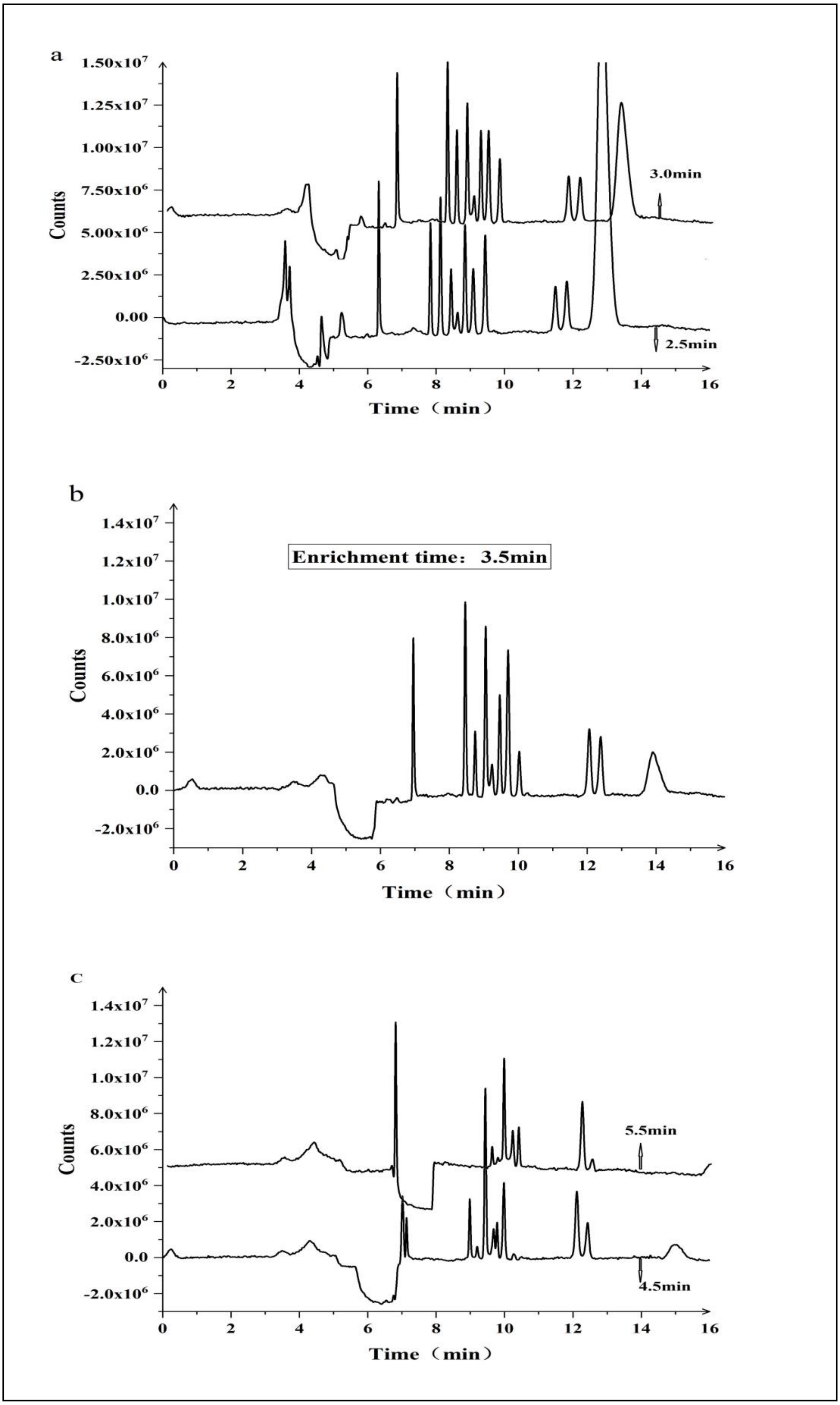

Figure S1. The chromatogram of different duration of the on-line extraction step

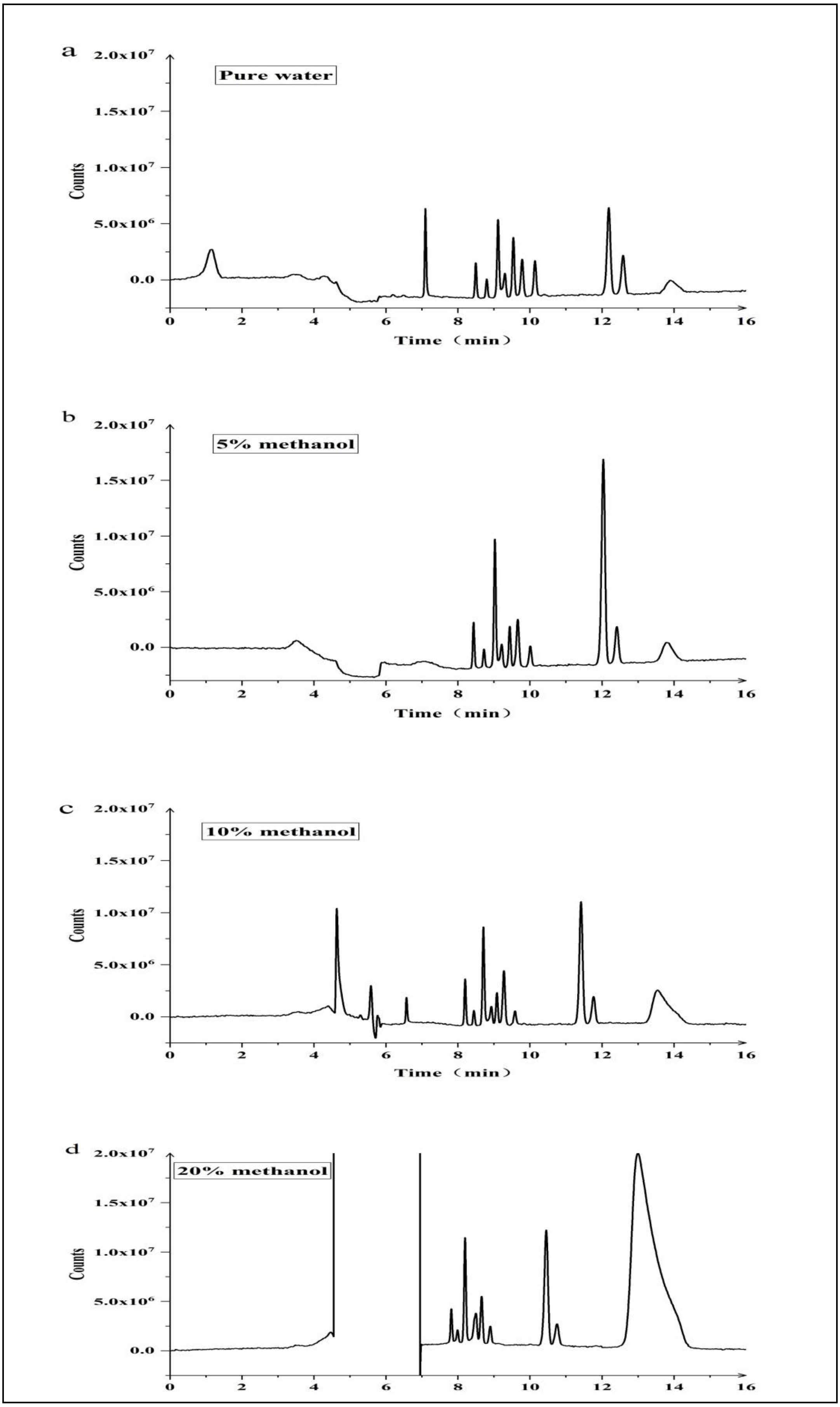

Figure S2. The chromatogram of different carrier mobile phase of the on-line extraction step

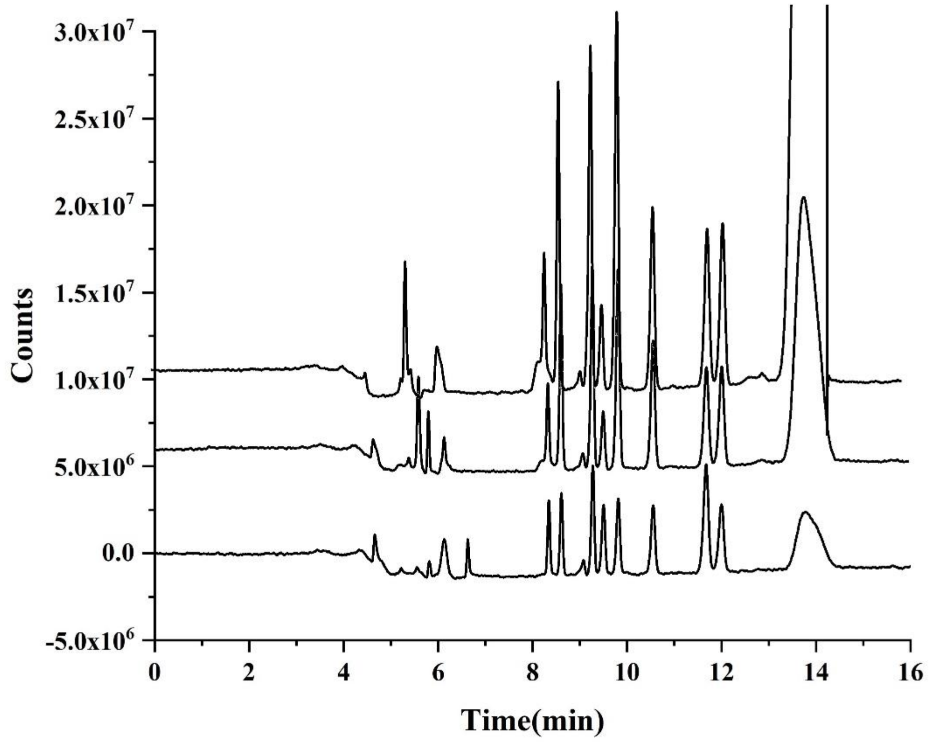

Figure S3. The chromatogram of different sample enrichment volume of the on-line extraction step

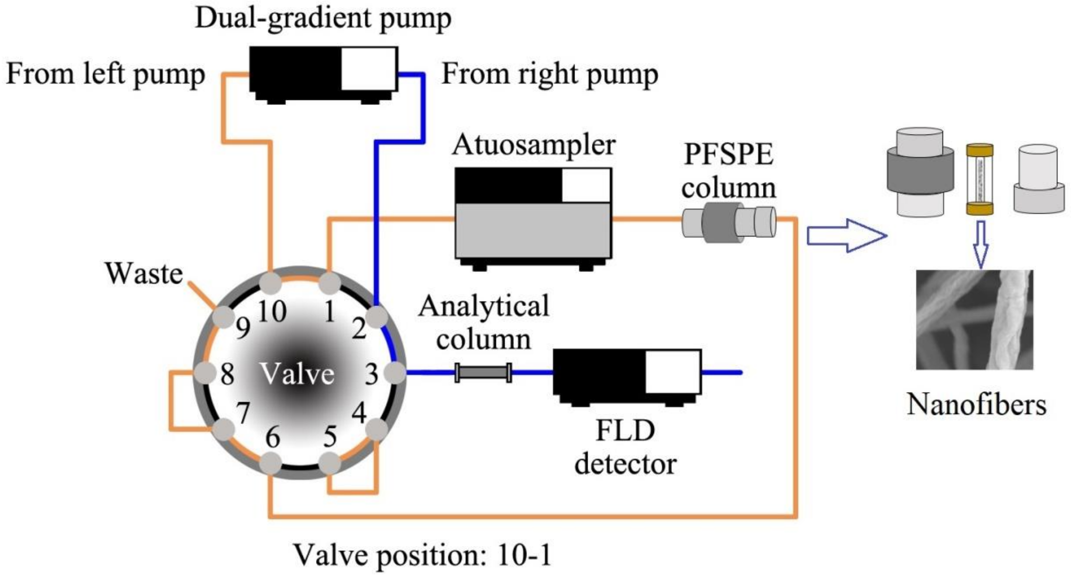

Figure S4. The preparation of the on-line PFSPE column and connection to the HPLC system

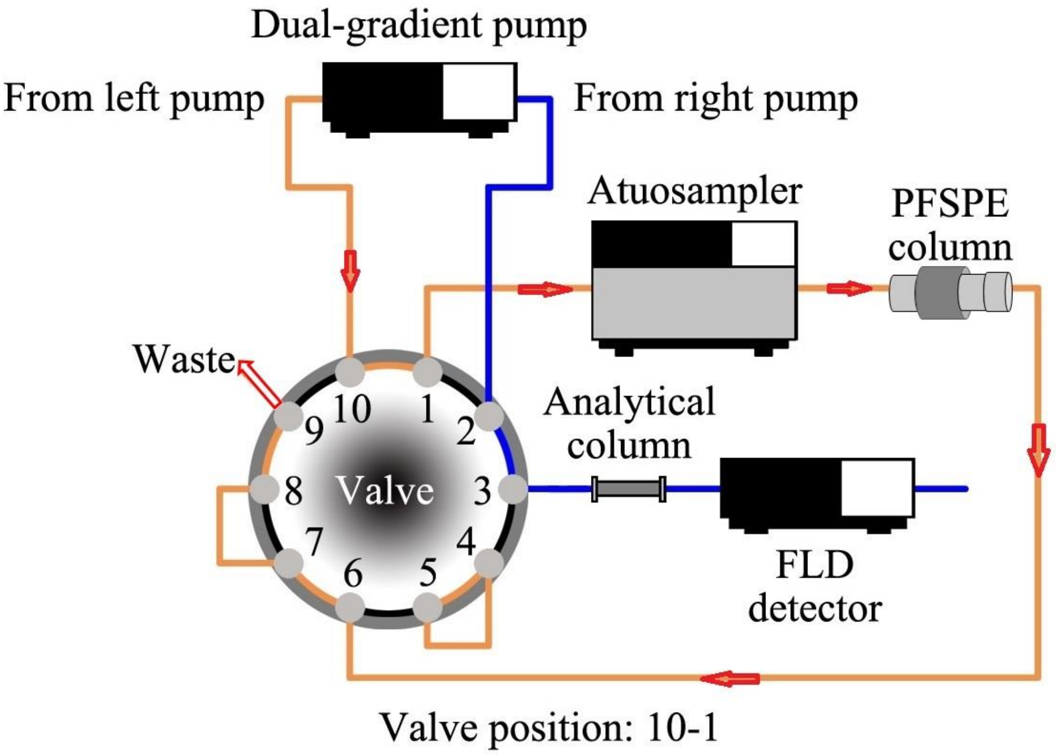

Figure S5. The schematic diagram of on-line sample pretreatment

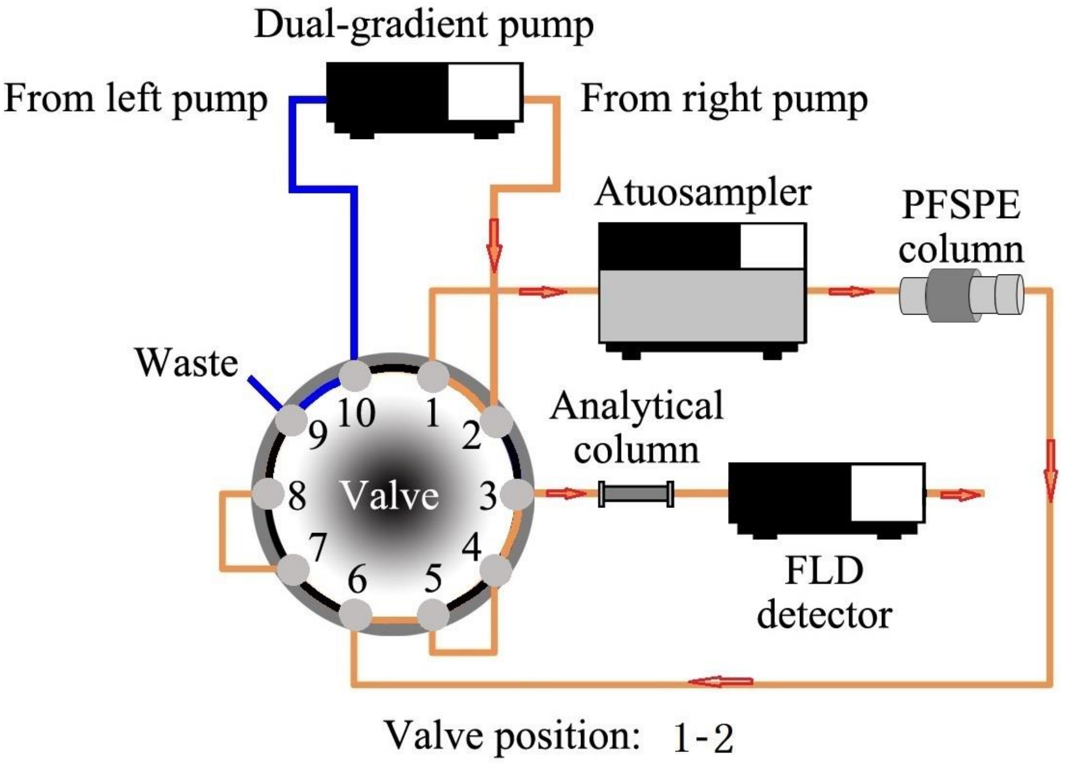

Figure S6. The schematic diagram of on-line transfer of the target compounds
